# Acceptability and usability of smartphone-based brainwave entrainment technology used by individuals with chronic pain in a home setting

**DOI:** 10.1177/2049463720908798

**Published:** 2020-02-21

**Authors:** Helen N Locke, Joanna Brooks, Laura J Arendsen, Nikhil Kurian Jacob, Alex Casson, Anthony KP Jones, Manoj Sivan

**Affiliations:** 1 Academic Department of Rehabilitation Medicine, Leeds Institute of Rheumatology and Musculoskeletal Medicine, University of Leeds, Leeds, UK; 2 Leeds Community Healthcare NHS Trust and Leeds Teaching Hospitals NHS Trust, Leeds, UK; 3 Manchester Centre for Health Psychology, The University of Manchester, Manchester, UK; 4 Division of Functional and Restorative Neurosurgery, Eberhard Karls University of Tübingen, Tübingen, Germany; 5 Department of Electrical and Electronic Engineering, The University of Manchester, Manchester, UK; 6 Human Pain Research Group, Division of Neuroscience and Experimental Psychology, The University of Manchester, Manchester, UK

**Keywords:** Mobile applications, alpha rhythm, EEG phase synchronisation, pain perception, pain management

## Abstract

**Background:**

Brainwave entrainment (BWE) using rhythmic visual or auditory stimulation has many potential clinical applications, including the management of chronic pain, where there is a pressing need for novel, safe and effective treatments. The aim of this study was to gain qualitative feedback on the acceptability and usability of a novel BWE smartphone application, to ensure it meets the needs and wishes of end users.

**Methods:**

Fifteen participants with chronic pain used the application at home for 4 weeks. Semi-structured telephone interviews were then carried out. A template analysis approach was used to interpret the findings, with an initial coding template structured around the constructs of a theoretical framework for assessing acceptability of healthcare interventions. Structured data analysis generated a final modified coding structure, capturing themes generated across participants’ accounts.

**Results:**

The four main themes were ‘approach to trying out the app: affective attitude and ethicality’, ‘perceived effectiveness’, ‘opportunity costs and burden’ and ‘intervention coherence and self-efficacy’. All participants were willing to engage with the technology and welcomed it as an alternative approach to medications. Participants appreciated the simplicity of design and the ability to choose between visual or auditory stimulation. All the participants felt confident in using the application.

**Conclusion:**

The findings demonstrate preliminary support for the acceptability and usability of the BWE application. This is the first qualitative study of BWE to systematically assess these issues.

## Background

Pain is a physiological marker of illness or injury and can be considered as a learning signal.^1^ However, when pain is chronic, it is associated with a range of physical and psychological pathologies.^2,3^ Chronic pain is a significant health problem affecting around 20% of adults in Europe^4^ and is one of the largest contributors to adult-onset disability worldwide.^5,6^ The cost associated with chronic pain is estimated to exceed that of cancer, diabetes or heart disease.^7,8^

The mainstay of treatment for chronic pain is with pharmacological therapies such as opioids and non-steroidal anti-inflammatory drugs (NSAIDs). Yet, evidence for the effectiveness of long-term opioid treatment is limited and NSAIDS are not recommended for long-term use. Moreover, both come with a risk of serious side effects.^9–13^ Thus, there is a pressing need for effective, safe and accessible alternatives for the treatment of chronic pain.

A target of considerable interest for the development of novel treatment approaches is the brain’s response to pain. Pain experience is the result of an integration of sensory pain information and various other influences including attention, emotions and expectations within a widespread brain network called the pain matrix.^14–16^ Chronic pain is associated with changes in brain structure and function^17–19^ and increased activity in brain regions involved in emotional processing of pain.^20^ Therefore, brain-stimulation techniques that can modulate these responses (‘neuro-therapies’) could be used to relieve pain.^21^

A particularly promising target for neuro-therapies is alpha activity–oscillatory brain activity at 8–12 Hz that can be measured using electroencephalography (EEG). Alpha activity has an active role in coordinating the processing of incoming sensory information, including sensory pain information. Moreover, frontal alpha in particular has been linked to functions of top-down control,^22^ attention^22,23^ and the modulation of pain perception by placebo-induced expectations of pain relief.^24^ Crucially, pre-stimulus alpha activity (directly before the onset of pain)^25–29^ is related to pain experience; higher alpha activity is associated with lower pain ratings and vice versa.^28,29^ A number of studies have also identified differences in resting-state alpha activity by comparing patients with chronic pain to pain-free controls.^30–34^ Importantly, a similar inverse relationship between alpha activity and chronic pain intensity has been found.^35^ Thus, stimulating the brain to increase alpha activity could lead to a reduction in chronic pain.

Rhythmic visual, auditory or transcranial alternating current stimulation (tACS) can be used to increase alpha activity non-invasively. When stimulation is applied at the alpha frequency (i.e. 8–12 Hz), this results in an enhancement of alpha activity via a mechanism of entrainment, whereby neural oscillations synchronise with the frequency of the stimulus. As more neurons synchronise, this is registered as an increase in alpha activity.^36^ Studies have demonstrated a reduction in experimental pain with alpha frequency tACS^37^ and with auditory and visual stimulation.^38,39^ More recently, a 2019 study has demonstrated first evidence that increasing alpha with tACS is also effective in relieving chronic pain.^40^

While these studies have shown positive results in an experimental setting, there is a clear need to translate this into a practical technology that can be used by patients for pain relief at home. For this purpose, we developed a technology for presenting alpha range visual and auditory stimulation integrated within a smartphone application (app). Although purpose-built goggles are used in a lab setting, the app can be used with a virtual reality (VR) headset for a similar experience. Headphones are used to deliver the auditory stimulation in the form of binaural beats, whereby the right and left ears receive tones at slightly different frequencies (e.g. 440 and 450 Hz) such that the perceived beat frequency is the difference between the two frequencies (e.g. 10 Hz).^41^

The design and development of this app must be guided by potential future users of the technology to ensure it meets their needs, as per the recent NICE Evidence Standards Framework for Digital Health Technologies (March 2019).^42^ Although the importance of assessing ‘acceptability’ is well established, the term has previously been criticised as ill-defined, under-theorised and poorly assessed.^43^ In this work, we therefore drew on a recently proposed Theoretical Framework of Acceptability (TFA)^43^ to structure our investigation. The concept of ‘usability’ is also captured within the framework to explore how easy to use, intuitive and accessible the app was for the users.

The aim of this study was therefore to gain qualitative feedback from patients with chronic pain regarding the acceptability and usability of a brainwave entrainment (BWE) smartphone app, to guide further design and development.

## Materials and methods

This study used qualitative methodology to explore and gain in-depth understanding of participants’ experiences and views.^44^

### Participants

Inclusion criteria:

- Adults aged 18 or older;
- Clinically significant pain (for which they have sought medical input) for more than 3 months;
- Able to consent to participation.

Exclusion criteria:

- Personal or first-degree relative history of epilepsy or convulsions/seizures;
- History of discomfort or eye twitching with flashing lights;
- Severe or frequent headaches;
- Current or planned hospitalisation during this study.

Sixteen participants were identified from local musculoskeletal, rheumatology and rehabilitation clinics. The first 14 participants also took part in a lab-based visual or tactile entrainment study with L.J.A., which measured the levels of entrainment with EEG while assessing the subjective pain scores. Although these studies used similar BWE technology, the participants’ first experience of using the BWE smartphone app was for this qualitative study. The subsequent two participants were recruited directly into this study by H.N.L. Participants were given a verbal explanation and a participant information sheet explaining the study. Written informed consent was obtained after 24 hours. One participant dropped out immediately after consenting.

Participants had a mean age of 47 years (range, 22–71 years) (Table 1). Of the 15 participants who completed this study, 14 (93%) owned a smartphone, while 13 (87%) stated that they used apps on a regular basis. Only one participant (7%) felt that they were not confident in downloading and using apps. Thirteen (87%) owned a laptop or personal computer, while six (40%) owned a tablet computer.

**Table 1.** Participant demographics and pain condition.

Participant no.	Age	Gender	Pain condition
1	23	F	CWP
2	51	M	CWP
3	62	F	CWP
4	50	F	Osteoarthritis
5	71	M	Cervical stenosis, osteoarthritis
6	39	F	CWP
7	47	F	Osteoarthritis
8	47	F	CWP
9	47	M	Musculoskeletal groin pain
10	22	F	CWP
11	35	F	CWP
12^a^	–	–	–
13	32	F	CWP
14	66	M	Cervical stenosis
15	48	M	Osteoarthritis and haemophilia
16	69	F	Radiculopathy

CWP: chronic widespread pain; M: male; F: female.

a Participant dropped out prior to data collection.

### The smartphone application

**Figure 1.**
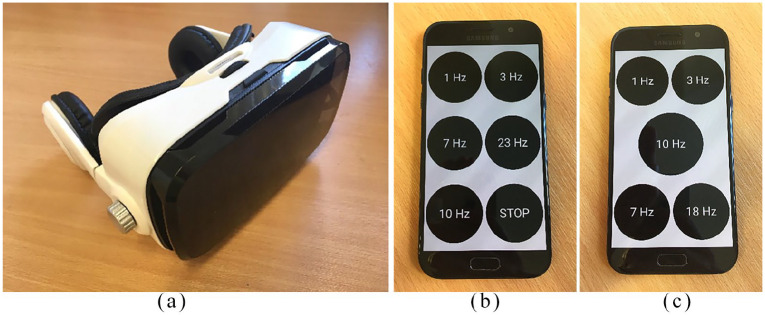
(a) Virtual reality headset, (b) smartphone showing frequency options for binaural beats (auditory) stimulation and (c) smartphone showing frequency options for visual stimulation.

The BWE smartphone app was developed by N.K.J. and A.C. as a research tool. On the home screen are options for ‘visual stimulation’ and ‘binaural beats’. Each option has a choice of four or five different frequencies, the lowest being 1 Hz and the highest being 23 Hz (for binaural beats) or 18 Hz (for visual stimulation) (Figure 1). Not all the frequencies are within the alpha range and it is therefore not expected that these would achieve alpha BWE; the recommended frequency setting for pain relief is 10 Hz based on preliminary evidence in healthy volunteers. However, having multiple frequency options within the app will allow the flexibility of testing different frequencies for pain relief in future efficacy trials.

The participants also had the option of choosing between visual and auditory stimulation as it was felt that allowing individuals to have a choice would make the study more inclusive, as some individuals with chronic pain can be intolerant to specific sensory modalities. This is an important aspect of user involvement in the iterative design and development process of the technology. The binaural beats option is designed for use with standard headphones or earphones. The visual stimulation option should be used with the phone placed inside a standard VR headset, such that it can be viewed directly in front of the eyes. Volume and brightness can be adjusted as per the participant’s preference using the phone settings.

### Study methods

At an individual face-to-face meeting with H.N.L., participants were provided with an Android phone, a VR headset and earphones to use for the entire duration of this study. Participants received a demonstration of the app and were given the opportunity to practice using it. Participants were encouraged to use the app at home at least once a day for 10 minutes in order to gain meaningful feedback. As there is no available data on BWE use longer than 40 minutes, it was suggested that they do not exceed this limit. Participants were provided with a contact email address and telephone number of the research team in case of any questions or issues with the app during the study period.

### Data collection

After 4 weeks, the participants were contacted by telephone for a semi-structured interview with H.N.L. lasting an average of 28 minutes (range, 16–55 minutes). An interview guide was developed by H.N.L. and J.B., based on the topics of technology literacy, the participant’s pain condition, experiences of using the app (usability, impact on activity, likes and dislikes) and smartphone technology in healthcare. Questions were structured around the interview guide, but the semi-structured approach allowed for exploration of relevant topical trajectories in the conversation (see Supplemental material). Interviews were recorded on a secure laptop using Audacity software.^45^ Recordings were then transcribed verbatim and any identifiable data were removed.

### Data analysis

Interview findings were analysed thematically using template analysis,^46^ a style of thematic analysis that provides a systematic approach to categorising qualitative data in hierarchical clusters. Template analysis permits the definition of ‘a priori’ themes – these are themes identified as likely helpful and relevant to the research focus, which are then refined and developed through analysis. Our coding template was structured around the TFA,^43^ a multi-faceted construct consisting of seven domains (in the context of this work, the term ‘intervention’ refers to use of the app in the home environment):

- Affective attitude (how an individual feels about an intervention);
- Burden (the perceived amount of effort it takes to engage with an intervention);
- Ethicality (the extent to which an intervention is congruent with an individual’s belief system);
- Intervention coherence (how an individual understands the aims of an intervention and how it works);
- Opportunity costs (the extent to which an individual needs to compromise existing benefits or values to engage with the system);
- Perceived effectiveness (how well an intervention achieves the desired outcomes);
- Self-efficacy (an individual’s level of confidence that they can engage in the behaviours required to participate with an intervention).

Analysis followed the procedural steps described by King and Brooks^47^ from what they describe as a qualitative neo-positivist position (for the purposes of this study, we have taken a realist approach that assumes an unproblematic relationship between our participants’ accounts and (a shared) external reality). Preliminary coding involved highlighting data of relevance from interview transcripts and assigning these segments to our a priori headings. Segments were then grouped into sub-themes to capture the finer details of participant accounts. Once a complete analysis template was constructed, this template was applied to the full dataset. Throughout analysis, the validity and consistency of coding was discussed between H.N.L., M.S. and J.B. and the final thematic framework was agreed by the full research team.

### Ethical considerations

Ethical approval for this study was gained from the Health Research Authority (IRAS Project ID 223210). This study was nested in an application for the wider Neuro-therapeutic Interventions for Pain (NTIP) Project by the Human Pain Research Group at The University of Manchester, which also included EEG and neurofeedback studies on the alpha brainwave response in chronic pain. Participants could withdraw from the study at any point up to data analysis without giving a reason.

## Results

The TFA defines acceptability as ‘a multi-faceted construct that reflects the extent to which people receiving a healthcare intervention consider it to be appropriate, based on anticipated or experienced cognitive and emotional responses to the intervention’.^43^ Table 2 shows our analysis template including all top-level themes (derived from the TFA) with sub-themes. Below we describe how participants’ accounts of using the BWE smartphone app mapped onto the TFA domains with illustrative quotes.

**Table 2.** Themes adapted from the theoretical framework with sub-themes from data analysis.

Themes	Sub-themes
1. Approach to trying out the app: affective attitude and ethicality	1.1. The impact of living with pain 1.2. Current management options
2. Perceived effectiveness	2.1. Extent of pain relief 2.2. Additional reported effects
3. Opportunity costs and burden	3.1. Fitting app use into everyday life 3.2. Negative effects of app use
4. Intervention coherence and self-efficacy	4.1. Understandings of and familiarity with technology 4.2. Intervention usability and suggestions for development

### Approach to trying out the app: affective attitude and ethicality

In the TFA, ‘affective attitude’ refers to how an individual feels about an intervention and ‘ethicality’ relates to how it fits into the individual’s personal value system, influenced by personal experience. In our analysis, these domains were merged into one theme describing individuals’ feelings about their pain condition and current treatment options, which influenced their attitudes towards and willingness to try this novel approach.

#### The impact of living with pain

Across participant accounts, the participants described physical restrictions placed on them by their pain condition. Participants also recounted isolation and social limitations – their world becoming ‘smaller’ in both a physical and social sense. Concurrent with this was a sense of increased (but reluctant) dependency on close others. Anxiety, stress and low mood were key features of the condition for many. Participants frequently described their pain condition in expressive terms such as fighting a daily ‘battle’ and there was a feeling of desperation to be free from pain and able to live a ‘normal’ life.

##### Illustrative quotes

**Table:** 

P3	. . . when you’re in that sort of vicious circle, you don’t realise just how small your life is getting. You stop doing things because it’s too much of a struggle, and you start adjusting everything you do to manage the pain
P13	. . . it doesn’t matter what you do, you’ll never find a cure, there’s no cure, there’s no pain meds, there’s no meds in the world that are going to make you better

#### Current management options

Participants reported that the main treatments offered to them were pharmacological therapies and that there was a lack of alternative options available. There was a common feeling of being stuck between a rock and a hard place with regard to efficacy and side effects of medication. Alternative treatment options were actively sought and almost all had already tried other treatment strategies, often despite significant personal expense. Five participants specifically voiced a willingness to try anything that may potentially help relieve their pain.

##### Illustrative quotes

**Table:** 

P7	. . . the constant ‘do I take pain killers and not be in pain, but then not be able to function again?’. You know . . . It’s not always an easy decision
P1	Right now they don’t have many options for you. They just say we can put you on medication and that’s it really. And that’s quite upsetting

### Perceived effectiveness

Whether an intervention is understood as effective contributes significantly to acceptability. In this case, participants were aware that they had been recruited on the basis of their pain condition, but were not informed of any potential effects that they may experience from using the app. However, it is worth noting that the last two participants were recruited after a press release, which included information about this technology that may have influenced their expectations. Notably, the perceived effectiveness of the app was not exclusively related to pain relief. Indeed, there were a range of ways in which participants interpreted the effectiveness of the app.

#### Extent of pain relief

This was a qualitative study focusing on usability and acceptability of the app design and therefore clinical efficacy cannot be ascertained based on this feedback. However, five participants did report a meaningful improvement in their pain symptoms after using the app. Others had more mixed views or were uncertain about the impact on their pain.

##### Illustrative quotes

**Table:** 

P15	It was miraculous. I reckon I’ve gone from, probably from reliant on anti-inflammatories to 80% less . . . Yeah, I’m really keen to know about the science a bit more and how it works, but at the moment I’m just happy feeling confident that it works
P9	I haven’t noticed any difference . . . some weeks are better than others but again I have no idea what that would be attributed to. I genuinely have good weeks and bad weeks

#### Additional reported effects

The most commonly stated effect was relaxation, stated by 10 out of the 15 participants, which was felt to be very positive even if the individual’s pain level remained the same. This was either the result of a direct ‘meditative’ effect from the rhythmic stimulation or resulted from taking the time out to quietly sit and relax. In addition to this sense of relaxation and restfulness, three participants reported falling asleep when using the app. However, it was less apparent whether there was any effect on overnight sleeping pattern.

##### Illustrative quotes

**Table:** 

P4	You could just go off into your own little world with it . . . It was comfortable, you could just relax into it and let time pass by quite happily
P11	I think the massive benefit for me as I said was almost like a calming meditative. That particular 7 Hz programme on the audio became almost like a calming, zoning out, meditative thing for me to use . . . for me that was a really fantastic positive effect
P5	I did them both [visual and auditory stimulation] lying on a sofa, with my head on a pillow on one arm and spread out across the sofa. So that was fine. In fact, once or twice I fell asleep it was that peaceful

### Opportunity costs and burden

The TFA defines ‘opportunity costs’ as the ‘extent to which benefits, profits or values must be given up to engage in the intervention’. ‘Burden’ is the perceived amount of effort it is likely to take to engage with an intervention. The two have been merged into one theme in our analysis, describing how the intervention fitted into participants’ daily lives and the impact on their activities, responsibilities or relationships.

#### Fitting app use into everyday life

Some participants found that they had no difficulty fitting the app into everyday life. However, others found it challenging to take time out from their existing responsibilities and felt uncomfortable doing so. Two participants felt that on occasion the psychological effort required to use the app was too great, due to the low mood and apathy associated with their condition. Three found it difficult to remember to use the app and felt this placed additional burden on them. Several participants suggested that the app could be modified to include a mechanism of receiving a reminder or prompt. Others expressed the view that if effective, then incorporating the app into your life is easy, as one would always make time to use something that is beneficial.

##### Illustrative quotes

**Table:** 

P4	It’s fine during the day when I’m at home on my own. But when you’re feeling a little bit of pain in the evening . . . to either go out of the room or stay in the living room with the rest of the family and saying ‘I’m just putting this on’, putting that on then didn’t seem to fit right with me
P15	Because I think my view is that, if you’ve got something that you’ve got a positive association with, using it isn’t negative and isn’t a hindrance
P2	Yeah. Umm there was some days I didn’t use it . . . there were certain days when I had a very low mood and you know it was like, you know, I couldn’t be bothered with anything
P1	But it crept down to once a week because it just became quite difficult to remember to do it. At first it’s a novelty, you do it a few times a week, then it’s hard to remember, so maybe a sort of reminder within the app would be good . . .

#### Negative effects of app use

Some participants reported negative effects when using the app. Five participants reported exacerbation of existing headaches or a new headache. Several described an increased sensory sensitivity associated with their condition, which affected their tolerance of the app. Overall, participants were positive about the VR headsets; however, two mentioned that the headset was heavy and lighter headsets would be preferable. Finally, participants also found that the all-encompassing nature of the stimulation, particularly with the visual stimulation via the headset, could be overwhelming and provoke anxiety.

##### Illustrative quotes

**Table:** 

P7	With the sounds I just used it for about 10 minutes because after that it tended to give me a bit of a headache. But that may have been again something to do with the volume
P6	I think for someone like me, maybe for someone who has other types of pain it might be helpful, but I think because mine is nerve related and already on those pathways, I’m feeling a lot of discomfort. You know, it just felt like it was exacerbating the pain
P10	It just made me feel a little bit anxious . . . It was mainly just the, how bright it was. I don’t really do well with flashing and things . . . It just makes me feel quite dizzy and anxious

### Intervention coherence and self-efficacy

This theme merges the TFA domains ‘intervention coherence’ and ‘self-efficacy’ and describes how participants understood the intervention, perceived barriers and enablers to using the app, what aspects of the app were most important to users and what might be modified to meet their needs.

#### Understandings of and familiarity with technology

Participants were interested in the concept of using technology as a condition management strategy and wanted to know more about the science behind the app. Participants talked in positive terms about ways in which the app could be individually tailored and provided a heightened sense of personal control of condition management.

Most participants were very comfortable around technology and used multiple technologies daily, including smartphones, laptops and tablets. They also used apps on a regular basis, with some having previously used apps for health or well-being. There was a recognition that smartphones are now very commonplace and an essential part of everyday life for many. However, participants recognised that this would not be the case for everybody, in particular those from the ‘older generation’.

##### Illustrative quotes

**Table:** 

P7	I think obviously I’m a very confident user of technology. But I think not everybody is. My mother-in-law for example, will only use the computer if somebody puts it in front of her. She wouldn’t dream of having a mobile phone
P15	It’s making me think about the science of what’s going on. And the brain side of things . . . It’s quite profound really
P10	It could have a really big impact on the way you feel about something if you feel like you’re actually helping yourself and you’re not relying on someone else to fix the problem

#### Intervention usability and suggestions for development

Feedback from early patient and public involvement workshops indicated that the app should be kept simple and straightforward. Participants in the study generally agreed with this sentiment and appreciated the app’s simplicity of design and ease of navigation. Participants were also asked about the VR headsets to gain an idea of whether using the VR headset would be a limitation for any users. Although most found it quite simple, some felt that it was ‘fiddly’ to use.

Visual and auditory stimulation had similar levels of acceptability among participants: six participants preferred the visual stimulation, five preferred the auditory stimulation, no preference was expressed by two participants and two tried only the visual stimulation. Reasons given for preferring the visual stimulation were that it was more immersive and it was easier to ‘zone out’ when using it. Reasons for preferring the auditory stimulation included finding the VR headset uncomfortable and finding the binaural beats more relaxing.

However, there was a range of preferences with regard to the frequency used. Interestingly, some individuals preferred the slower frequencies, finding them more calming or less intense. Contrastingly, others preferred the higher frequencies, commenting that they perceived more of a stimulatory effect from the faster speeds, and thus felt it was more likely to relieve their pain.

##### Illustrative quotes

**Table:** 

P5	Yeah it was so easy to use, you know, you just had to tap the screen . . . it was just brilliant, brilliant
P11	Always a slower speed would be a preference, because for me psychologically I just found it calmer and the same with the sounds as well
P8	I preferred the higher ones if I’m honest . . . I just seemed to get better effects from them

## Discussion

This study demonstrates preliminary support for the acceptability and usability of the BWE app for users with chronic pain.

There is increasing awareness of the need to address acceptability in the development of new healthcare interventions. Users gain greater clinical benefit from treatments they consider to be acceptable, as they are more likely to use the treatment as advised.^43^ Surprisingly, the perceived effectiveness of the app was not related to pain relief alone, but benefits such as relaxation and ‘zoning out’ were considered as equally positive results.

The app provides a self-directed tool for BWE in the home environment. Previous studies have demonstrated that using a healthcare app can empower patients by providing a means to self-manage their condition.^48^ It is increasingly recognised that complex health conditions such as chronic pain require personalised treatments, and effective ‘e-health’ technologies, such as smartphone apps, can facilitate this.^49^ This is supported by the European Commission’s e-Health Action Plan.^50^

The home-based nature of the app is likely to be more acceptable to patients who struggle to engage in activities outside the home environment. Given the largely negative attitudes towards pharmacological treatments among participants, there were clearly articulated wishes for alternative approaches to pain management. Participants were keen to emphasise their willingness to try a novel approach and their openness to use the app (although expectations about cure or control of symptoms may need careful management). Smartphone-based technologies are increasingly used as healthcare tools, as they are accessible and affordable. Although there is some discrepancy in smartphone use between age groups and social classes, the trend of number of users has been steadily increasing among all user groups.^51^ It is therefore an effective means of targeting groups that may traditionally struggle to access healthcare.

The term ‘usability’ is used in a broad sense in the context of the TFA to encompass the users’ perspectives on how easy to use, intuitive and accessible they found the app. However, there have been many proposed definitions of usability in the context of software development,^52,53^ including the international standard ISO 9241-11:2018, which focuses on effectiveness (the ability of users to achieve the intended goal), efficiency (resources consumed in achieving the intended goal) and satisfaction (referring to how users feel).^52,54^ While this study did not use metrics to measure the above factors, nonetheless an interpretation can be made based on qualitative user feedback. A fundamental aspect of this study design was recruiting participants with chronic pain, who are the intended end users of the intervention, and setting the study in the home environment. This was crucial in assessing the usability of the app in the intended context of its clinical application.^53^

Participants generally found the app to be easy to use. All participants were able to use both the auditory and visual stimulation at the different frequency settings and responded positively to its simple design. A ‘user-friendly interface’ is an essential feature of an effective health app according to Higgins in his 2016 commentary on smartphone apps for health and fitness.^55^ Perhaps in contrast to this were the suggestions from participants to incorporate other features, such as reminders or pain monitoring, which may complicate the design. However, such features have been used effectively in other health and wellness apps.^56,57^

Additionally, participants were able to use the VR headset with little difficulty. However, several participants commented on the fiddly nature of placing the phone within the headset. The key usability goal was to ensure that the intervention is accessible to all potential future users and this may be an issue for users with dexterity difficulties. One of the recognised limitations of health and wellness apps is that they may not always be suitable for users with different needs or disabilities.^55^ A possible solution to this is a bespoke set of goggles, which may also be lighter and more comfortable for users. Another recognised limitation is the need for active engagement from the user.^55^ This was echoed by the participants, who found that at times they lacked the energy or will to use the app. This is likely to be a common difficulty for those with chronic pain or mood disorders and should be taken into consideration.

As well as the positive effects mentioned above, some participants experienced mild headaches or discomfort when using the app. In some cases, this was felt to be linked to their pain condition. No similar effects have previously been reported.^38,39^ However, this is the first study of its kind using BWE in the home setting on a regular basis. These adverse effects should be taken into consideration when applying the intervention in clinical settings.

As a qualitative study, findings cannot be used as a means of assessing clinical efficacy. However, the semi-structured interview process allowed for full exploration of what was deemed to be important to each participant, providing rich and useful data. One limitation was that the interviews were all carried out by a single researcher with a clinical background. It is possible that different ideas may have arisen if the interviewer had been from an app development background, for example. However, the study was performed in collaboration with professionals from a range of disciplines including engineering, psychology and pain medicine, who contributed at each stage. Analysis was carried out by one researcher, but regularly reviewed and discussed with other members of the research team and the final thematic framework was agreed by the full research team.

Moving forward, this study will guide the further development of the BWE app. Alongside this, further efficacy studies will be warranted, using quantitative measures of pain response to the app in the home setting. Dose response and duration of effect should also be established, as well as understanding the variability of response between individuals. Understanding the phenotype of responders versus non-responders may enable clinicians to target appropriate individuals for whom it is likely to be effective in future.

Although specifically used in the context of chronic pain in this study, BWE has the potential for a range of clinical applications in fields such as sleep and mood disorders. These findings are therefore applicable to other health contexts. Despite the increasing prevalence of healthcare apps, they are rarely developed collaboratively with input from app developers, healthcare professionals and patients.^58^ This study therefore adds to our understanding of what is important to end users of such technologies and may provide useful insight for the development of other healthcare apps for this patient group.

## Conclusion

BWE technology has been a recent focus of interest in the context of managing chronic pain. This technology can be delivered easily and affordably through a smartphone app. This study presents preliminary evidence for the acceptability and usability of the app in the home setting. While studies are ongoing into the clinical efficacy and dosing response of BWE in pain, this study provides the user perspective to ensure that the design and functionality of the app meet the needs and wishes of users with chronic pain.

## Supplemental Material

Qualitative_interview_guide_V4.1 – Supplemental material for Acceptability and usability of smartphone-based brainwave entrainment technology used by individuals with chronic pain in a home setting Click here for additional data file. Supplemental material, Qualitative_interview_guide_V4.1 for Acceptability and usability of smartphone-based brainwave entrainment technology used by individuals with chronic pain in a home setting by Helen N Locke, Joanna Brooks, Laura J Arendsen, Nikhil Kurian Jacob, Alex Casson, Anthony KP Jones and Manoj Sivan in British Journal of Pain
