# Supplementary material for: Acceptability and usability of smartphone-based brainwave entrainment technology used by individuals with chronic pain in a home setting
Source: Br J Pain. 2020 Feb 21;14(3):161–70. doi: 10.1177/2049463720908798 (PMC7453483; doi:10.1177/2049463720908798)
Supplement: Qualitative_interview_guide_V4.1 – Supplemental material for Acceptability and usability of smartphone-based brainwave entrainment technology used by individuals with chronic pain in a home setting [file Qualitative_interview_guide_V4.1.docx]

**Neuro-Therapeutic Interventions for Pain (NTIP) Project Phase 2: Feedback on smartphone pain diary and sensory stimulation apps.**

**Qualitative semi-structured interview guide.**

1. **BACKGROUND INFORMATION**

*Clinical condition*

Would you mind telling me a little bit about your pain condition? (symptoms, perceived cause, timeline, current treatment/management strategies/sleep/mood problems)

*Technology literacy*

What smartphone or other app enabled device do you use?
How often do you use apps on your phone or other device?
Have you used apps for health before?
Do you use any music/meditation techniques that involve using a smartphone app?
Do you feel confident in using a smartphone or other device to download and use apps?

1. **FEEDBACK ON SENSORY STIMULATION APP**
   1. **Experiences of using the app**

Could you tell me about how you found it to use the app? *(What did/didn’t you like?)*

With regards to the visual stimulation:

- How did you find this? *(What did you/didn’t you like?)*
- Did you notice any effects on your pain levels?
- Were there any other positive or negative effects that you noticed?
- How did you find using the headset? *(What did you/didn’t you like?)*

With regards to the auditory stimulation:

- How did you find this? *(What did you/didn’t you like?)*
- Did you notice any effects on your pain levels?
- Were there any other positive or negative effects that you noticed?

Which did you prefer to use and why?

How long did you use the app for each time?

Did you use any different frequency settings within the app? How did you feel about these? *(What did/didn’t you like? Which did you prefer and why?)*

Did you notice any other positive/negative effects at any of the frequencies? Did you notice any impact on your sleep?

How regularly did you use the app? How many days/weeks did you use the app for?

- 1. **Impact on activity**

Did using the app interfere with your daily life or activities in any way? *(In what way? Any specific examples?)*

Did using the app change how you engaged in activities in any way?

- 1. **Impact on cognitions**

Did using the app make you think about your symptoms any differently? *(How? Any specific examples?)*

- 1. **Other impressions**

Is there anything else you’d like to tell me about your experiences of using the app which we haven’t covered?

Overall, how useful do you think the app is?

1. **VIEWS ON SMARTPHONE TECHNOLOGY IN HEALTHCARE**

How would you feel about using healthcare apps alongside other treatments?

What would make you more likely/less likely to use this kind of technology?

What do you see as the main barriers for people using this type of technology?

Why did you choose to take part in this study?
